# Effects of a Teacher-Training Violence Prevention Program in Jamaican Preschools on Child Behavior, Academic Achievement, and School Attendance in Grade One of Primary School: Follow up of a Cluster Randomized Trial

**DOI:** 10.3389/fpsyg.2021.652050

**Published:** 2021-06-03

**Authors:** Helen Baker-Henningham, Yakeisha Scott, Taja Francis, Susan P. Walker

**Affiliations:** ^1^School of Psychology, Bangor University, Bangor, United Kingdom; ^2^Caribbean Institute for Health Research, University of the West Indies, Kingston, Jamaica

**Keywords:** violence prevention, teacher-training, conduct problems, social skills, early childhood, academic achievement, school attendance, low- and middle-income countries

## Abstract

**Objective:** We evaluated the effect of a universal, teacher-training, violence-prevention program implemented in preschool, on high-risk children's behavior, achievement, and attendance in grade one of primary school.

**Methods:** A cluster-randomized trial was conducted in 24 preschools in Kingston, Jamaica. Three children from each class with the highest level of teacher-reported conduct problems were recruited for evaluation of outcomes (*n* = 225 children). For this study, to increase power, we recruited an additional two children from each class with the next highest teacher-reported scores for conduct problems in preschool. In the final term of grade one of primary school, we assessed children's: (1) conduct problems and social skills at home and school, (2) academic achievement, language, and self-regulation skills, and (3) school attendance.

**Results:** 214/225 (95.1%) of the children evaluated in preschool were assessed in grade one of primary school; an additional 150 children were recruited to give 364 children (181 intervention, 183 control). Significant benefits of intervention were found for child academic achievement (Effect size (ES) = 0.23, *p* = 0.02), oral language (ES = 0.28, *p* = 0.006), self-regulation (ES = 0.25, *p* = 0.007), and school attendance (ES = 0.30, *p* = 0.003). No significant benefits were found for observed conduct problems (ES = −0.13, *p* = 0.16), and parent-reported conduct problems (ES = 0.10, *p* = 0.31) and social skills (ES = −0.07, *p* = 0.52). Benefits to teacher-reported conduct problems and social skills were significant at *p* < 0.1 (ES = −0.16, *p* = 0.09, and ES = 0.19, *p* = 0.06, respectively).

**Conclusion:** A scalable intervention involving training preschool teachers in classroom behavior management and how to promote child social-emotional competence led to positive outcomes in primary school across multiple child developmental domains for high-risk children.

## Introduction

Disruptive behavior disorders (DBDs) include conduct disorder and oppositional defiant disorder are one of the most common childhood mental health problems with a global prevalence of 5.7% (Polanczyk et al., 2015). Conduct problems are more common affecting 7–25% of young children and place children at increased risk for developing later DBDs and for academic underachievement, school dropout, drug use, and crime and violence in adulthood (Webster-Stratton and Hammond, 1998; Scott, 2015). Preventative interventions in early childhood are recommended to prevent the development of serious DBDs (Webster-Stratton and Taylor, 2001). Universal interventions to prevent DBDs are non-stigmatizing, often address multiple risk and protective factors thus leading to benefits across child developmental domains, and have potential for population-level improvements in child functioning as all children are exposed to intervention (Greenberg and Abenavoli, 2017). Universal, school-based, violence-prevention programs have been shown to reduce children's aggressive and disruptive behaviors (Hahn et al., 2007; Wilson and Lipsey, 2007), with some evidence of sustained benefits in adulthood (Hawkins et al., 2008). Common approaches used in these programs involve training teachers in classroom behavior management and/or how to promote children's social-emotional skills. Meta-analyses of classroom behavior management and social-emotional learning programs report benefits to multiple child outcomes including children's behavior, social-emotional skills, and academic skills with significant concurrent benefits across all domains and some evidence that benefits are maintained over time (Korpershoek et al., 2016; Mahoney et al., 2018). Meta-analyses of such interventions in early childhood educational contexts also report benefits to child behavior and social-emotional competence, (Schindler et al., 2015; Werner et al., 2016) with strongest effects from programs with an explicit focus on child social and emotional skills (Schindler et al., 2015).

Despite this large evidence-base for the effectiveness of school-based violence prevention programs for reducing child aggressive and disruptive behaviors and increasing child competencies, there are few trials from low- and middle-income countries (LMIC) (Burkey et al., 2018). This is a concern as: (1) almost 90% of the world's children and adolescents live in LMIC (Keiling et al., 2011), (2) many schools in LMIC have low levels of resources and are staffed by undertrained teachers, and we need evidence that programs can work in these low-resource contexts, (3) risk factors for conduct problems including violence against children by parents and teachers are widespread in LMIC, (Hillis et al., 2016; Gershoff, 2017), and (4) approaches used in high-income countries are often resource intensive and unlikely to be affordable.

We evaluated a teacher-training, violence prevention program in Jamaican preschools that involved training teachers in classroom behavior management and how to promote young children's social and emotional skills. Large and significant benefits were found for teacher practices and the classroom atmosphere at post-intervention and at 6-months follow-up (Baker-Henningham and Walker, 2018). Intervention teachers used more positive and fewer negative strategies with the whole class [mean effect size (ES) = 2.32 SD at post-intervention, 1.84 at follow-up], and with children with high levels of conduct problems on recruitment (mean ES = 0.67 at post-intervention only), and benefits were found for observer ratings of class-wide child appropriate behavior and interest and enthusiasm in learning activities (mean ES = 0.86 at post-intervention, 0.64 at follow-up). Importantly, these benefits were accompanied by significant benefits to conduct problems and social skills at school (mean ES = 0.56) and at home (ES = 0.22), and to school attendance (ES = 0.30) for children with heightened levels of conduct problems at baseline (Baker-Henningham et al., 2012). In the present study, we evaluated whether this preschool teacher-training, violence-prevention programme led to sustained benefits to child outcomes, for children with heightened levels of conduct problems at baseline, when children transitioned to primary school. Specifically, we investigated the effect of the preschool teacher-training program on child conduct problems, social skills, school attendance, school achievement, oral language, and self-regulation skills in the final term of grade one of primary school.

## Methods

### Study Design and Participants

The teacher-training, violence-prevention program was evaluated in a cluster-randomized trial in 24 community preschools situated within three educational zones located in disadvantaged, inner-city areas of Kingston and St. Andrew, Jamaica. Community preschools cater to children aged 3–6 years and are provided through community organizations, usually churches, with oversight from government. Over 98% of 3–6-year-old Jamaican children attend an early childhood educational institution, with the majority (over 75%) attending community preschools. All preschools within the three zones were surveyed and those meeting the inclusion criteria were invited to participate. Inclusion criteria were: (1) at least 20 children per class, (2) three to four classes of children, and (3) all teachers consent to participate in the trial. Fifty schools were approached and 24 preschools met all inclusion criteria and were recruited into the study (twenty-six schools were excluded: seven with <3, or more than four classrooms; 18 with <20 children per class; one refusal). In all preschools, children were grouped in same-age classrooms (3, 4, and 5-year-olds). The community preschools were staffed mostly by paraprofessional teachers, and had poor structural conditions, and few resources (Baker-Henningham et al., 2012; Baker-Henningham and Walker, 2018).

Children with heightened levels of conduct problems at baseline were recruited into the evaluation as school-based preventative interventions have been shown to benefit high-risk children the most (Wilson and Lipsey, 2007). Pre-school teachers rated all children in their class on a 10-question screen for conduct problems using a four-point scale (not true, just a little true, pretty much true, very true). Questions were based on age-appropriate items for a diagnosis of conduct disorder from the ICD-10 Classification of Mental and Behavioral Disorders: Diagnostic Criteria for Research (World Health Organization, 1993), (loses temper, back chats, disobedient/breaks rules, annoys others, blames others, easily annoyed, often angry, spiteful to others, fights or bullies, destroys property). Three children from each class, with the highest level of conduct problems were selected for evaluation. Exclusion criteria for children were: (1) school attendance <70%, (2) sibling of an enrolled child, (3) had a developmental disability, and/or (4) lived in an institution. Twenty-four high-scoring children were excluded and replaced by the next highest-scoring child in their class.

**Figure 1:**
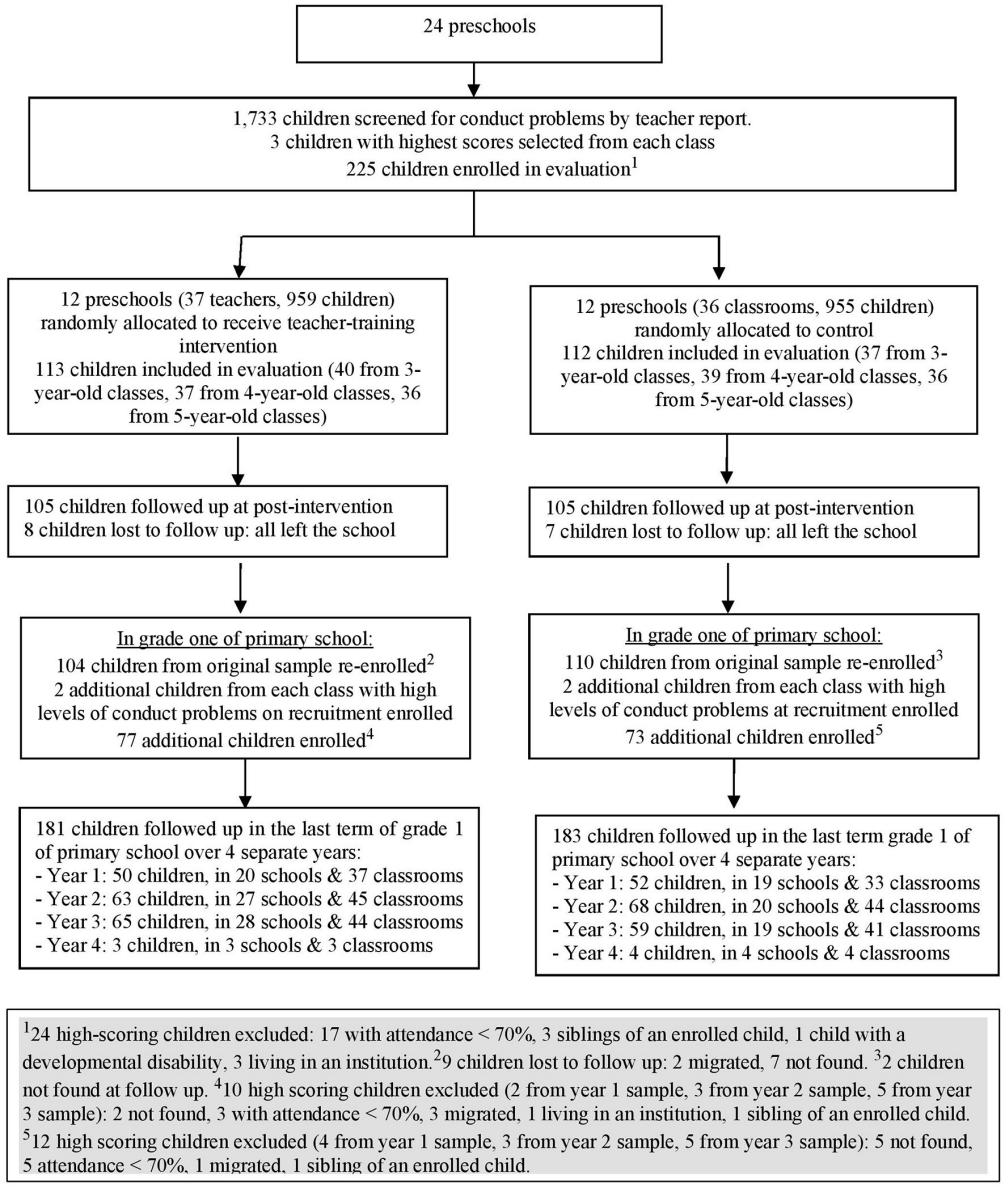
Study profile.

A total of 225 children were recruited from the 24 preschools at baseline and after randomization, 113 children attended preschools allocated to intervention and 112 children attended preschools allocated to control; 210 children were evaluated post-intervention (Figure 1). For the current study, we tried to locate all 225 children recruited at baseline. In addition, to increase power to detect significant differences between the groups, two additional children per class with the next highest levels of teacher-reported conduct problems on the 10-question screen at baseline were selected. Exclusion criteria were the same as for the original sample. Twenty-two high scoring children were excluded (10 intervention, 12 control), and were replaced by the next high-scoring child in the class (Figure 1). A total of 150 additional children (77 intervention, 73 control) were selected giving a total sample size of 364 children (181 intervention, 183 control).

For the current study, we report cross-sectional data collected when children were in the final term of grade one of primary school. Children transition to primary school at age 6 years and the data for this study was collected over 4 years until all children that were screened in preschool had transitioned to primary school.

The University the West Indies Ethics Committee approved the study (approval number: ECP148,10/11). Written informed consent was obtained from all school principals, teachers and parents of the selected children.

### Intervention

The intervention involved training all teaching staff and principals in intervention preschools in an adapted version of Incredible Years (IY) Teacher Training Program (Webster-Stratton, 2000). The core content of the program included: (1) developing positive teacher-child relationships, (2) using praise and rewards, (3) preventing and managing child misbehavior, and (4) teaching social and emotional skills. Teachers attended eight full-day teacher-training workshops and received four 1-h sessions of in-class support, conducted monthly for 4 months. Workshops included videotape and live modeling, practice activities, and discussions. In-class support sessions included modeling the use of the strategies, prompting the teachers to use them, providing supportive feedback, and collaborative problem-solving. The in-class support was designed to ensure teachers could utilize the strategies successfully in their individual classroom context and help teachers to generalize their use over the school day. Teachers were given practical classroom assignments after each workshop to encourage use of the strategies taught and were provided with key resources required to implement the program (including visual aids, stickers, a small hand puppet, and behavior planning forms). Adaptations to the IY program included: (1) adding locally developed materials and activities (for example, video vignettes, instructional materials, classroom assignments, lesson plans, visual aids), (2) lengthening the program from 5–6 days to 8 days, (3) including additional practical activities and small-group activities in training workshops, (4) providing in-class support, and (5) designing new content and placing increased emphasis on building positive relationships with children, being proactive to prevent behavior problems, and integrating activities to promote children's social-emotional competence into everyday teaching and learning activities. The intervention was delivered as intended, teachers reported high levels of satisfaction with the training and teacher attendance was high (Baker-Henningham and Walker, 2018). Full details of the intervention and the adaptations made have been described previously (Baker-Henningham and Walker, 2018). Preschool teachers in control schools did not receive the teacher-training workshops, in-class support sessions, or intervention materials. All study schools received a set of educational materials including blocks, manipulatives, and play doh.

### Measurements

Outcome measurements included child: (1) conduct problems by observation, teacher-, and parent-report, (2) social skills by teacher- and parent-report, (3) academic achievement and oral language skills by direct testing, (4) self-regulation through ratings during the test, and (5) school attendance from school records (see Supplementary Table 1). All outcome measurements have been used previously in Jamaica (Baker-Henningham et al., 2007, 2012) and were collected by data collectors (DCs) masked to group assignment. Teachers were also unaware of children's group assignment. DCs were rotated across classrooms and schools and conducted equal numbers of measurements with each group. Measurements were conducted in the final term of the school year when the children were in grade one of primary school.

#### Observations of Child Behavior

Children were observed for 5-min intervals, for 30 min a day, over two school days to give a total of 1 h of observation. When there was more than one target child in a class, children were observed for 5 min each on a rotational basis with a maximum of three children observed at one time. When there was only one target child per class, the child was observed for 5 min out of every 10 min. Event sampling was used to record each discrete act of aggressive/destructive behavior (e.g., hitting, throwing objects) and expressed as frequency per hour. Disruptive behavior (e.g., shouting, out of seat) was measured by recording whether the behavior occurred or not at 15 s intervals with a maximum possible score of 240. After each 5-min interval, observers also rated child behavior on four 7-point rating scales measuring the frequency and intensity of child conduct problems (e.g., displaying anger/frustration, non-compliance), activity level (e.g., inappropriate gross-motor and fine-motor movements), on-task behavior (e.g., engagement in learning activities), and follows classroom rules/expectations (e.g., obeys rules/follows routines). The mean score over 12 5-min intervals was used in the analyses. Higher scores indicate more of the characteristic. All behaviors were defined in a manual and were based on observational assessments used previously in preschools, operationalized for the primary school environment (Baker-Henningham et al., 2012).

#### Teacher- and Parent-Reported Child Behavior

All questionnaires were interviewer-administered. For teacher-reported behavior, we used the Sutter-Eyberg Student Behavior Inventory (SESBI) frequency scale (Rayfield et al., 1998) to measure child conduct problems and the School Social Behavior Scales-Social Competence Scale (SSBS) (Merrell, 2002) to measure child social skills. For parent-reported child behavior, we used the Eyberg Child Behavior Inventory (ECBI) frequency scale (Eyberg and Ross, 1978) to measure child conduct problems and the Strengths and Difficulties (SDQ) Prosocial Scale (Goodman, 1999) to measure child social skills. All measures had good internal consistency (Cronbach's alpha: median 0.87, range 0.71–0.97) and test-retest over 2 weeks (ICC: median 0.88, range 0.75–0.97) (Supplementary Table 2).

#### Child Academic Achievement, Oral Language, and Self-Regulation Skills

Reading and spelling were measured with the Letter-Word Identification, Passage Comprehension, and Spelling subscales of the Woodcock-Johnson III Tests of Achievement (Woodcock et al., 2001). Maths was measured with the Calculation and Reasoning and Concepts subscales of the Woodcock-McGrew-Werder Mini-Battery of Achievement (Woodcock et al., 1994). Receptive and expressive oral language skills were measured using the Understanding Directions and Story Recall subscales of the Woodcock-Johnson III Tests of Achievement (Woodcock et al., 2001). Test-retest over 2 weeks were ICC: median = 0.97 for child academic achievement tests and ICC = 0.82 for child language skills (Supplementary Table 2).

Children's self-regulation during the testing session was rated using ten 4-point scales from the Preschool Self-Regulation Assessment (PSRA) (Smith-Donald et al., 2007). Five items rated child attention (pays attention, careful, concentrates, daydreams, distracted) and five items rated child impulse control (thinks and plans, refrains from touching testing materials, does not interrupt tester, difficult waiting, remains in seat). Negative items were reverse coded and scores from the 10 scales were summed to form a total score with a minimum score of zero and a maximum of thirty. Internal reliability was Cronbach's alpha = 0.88, and test-retest over 2 weeks was ICC = 0.85.

School attendance for the first two school terms in grade one was calculated from classroom registers and expressed as a percentage.

#### Procedure and Quality Control

Ten DCs collected the outcome data for this study. Three DCs conducted observations of child behavior at school, three DCs conducted teacher interviews and child tests, and two DCs conducted parent interviews. Teacher questionnaires and child tests were administered at school and parent questionnaires at home. Child observations were conducted over two school days in each classroom. Only one child observer was present in a class at a time, with a maximum of two child observers present in a school. Child tests and teacher interviewers were conducted after all child observations in a classroom were completed. DCs were trained over a 3–4-week period prior to each year of data collection including 1-week in-office training, 1–2-weeks field training, and 1-week field reliabilities. Inter-observer reliabilities were calculated between the trainer and each DC prior to data collection and for a minimum of 10% of measurements during ongoing data collection. For child observations, interobserver reliabilities were calculated for 5-min observations intervals and the intraclass correlation coefficients (ICC) were median 0.93 (range 0.90–0.95) prior to data collection, and ICC = 0.93 (0.84–0.97) during the study (Supplementary Table 3). For child tests and teacher and parent interviewers, ICCs were >0.95 throughout.

### Statistical Analysis

For the sample size calculation, we assumed an average of sixteen children per cluster, and an intra-cluster correlation coefficient of 0.05. With a minimum of 175 children per group, we could detect an effect of 0.4 SD, with 80% power and at a 0.05 level of significance.

All variables were checked for normality. Multilevel multiple regression analyses were used to determine the effect of intervention on child outcomes to take into account the clustered nature of the data. Exploratory factor analysis of the observed child behavior variables produced one factor; factor analysis of the academic achievement test scores produced one factor, and factor analysis of the oral language test scores produced one factor (Supplementary Table 4). The factor scores for these three outcomes were saved as regression scores and used in the analyses (DiStefano et al., 2009). All other outcomes (parent and teacher-reported conduct problems and social skills, child attendance, and self-regulation) were checked for normality and then standardized. Self-regulation, parent-reported prosocial behavior and school attendance were positively skewed and were transformed by squaring prior to standardization. The use of factor scores and standardized scores resulted in regression coefficients expressed in standard deviations for all outcomes. In all analyses, child age and sex, dummy variables for data collector, dummy variables for the year of data collection, a variable for whether the child was evaluated in the original study or not, and group assignment were entered as fixed effects and school and classroom as random effects. Multilevel analyses were conducted with MLWin version (v3.05) (Charlton et al., 2020).

## Results

### Sample Characteristics

We identified 214/225 (95.1%) of the children recruited in preschool (104/113 intervention, 110/112 control). Two children lost to follow-up had migrated (both intervention), and nine were not found. There were no significant differences between those lost and those found on family characteristics and child behavior at home and at school at baseline. However, children lost to follow up were younger (*p* = 0.02) and less likely to be male (*p* = 0.08) (Supplementary Table 5).

Over the 4 years of the study, study children were dispersed over fifty primary schools and 149 different classrooms. There was a mean of 6.25 children per school, with a range from 1 to 57. Over 54% (198/364) of the children attended the same five primary schools [54.1% (98/181) intervention, 54.6% (100/183) control]. In addition, 49 classrooms in 14 schools catered to nearly 50% (177/364) of the children [47.5% (86/181) intervention, 49.7% (91/183) control].

There were no significant differences between the groups on child, family, teacher and classroom characteristics in grade one, and for the children evaluated in preschool, no significant differences between the groups at baseline (Table 1).

**Table 1:** Child and family characteristics and child behavior in preschool by study group. Values are Mean (SD) unless otherwise stated.

**Children evaluated in preschool**	**Intervention*n* = 113**	**Control*n* = 112**	*P*-value*
**Child and family characteristics**			
Child age (in years)	4.2 (0.9)	4.2 (0.8)	0.86
Child sex: *n* (%) boys	67 (59.3)	71 (63.4)	0.53
Caregiver age	31.5 (10.6)	30.8 (8.7)	0.22
Caregiver finished high school *n* (%)	46 (40.7)	47 (42.0)	0.85
Father lives with child *n* (%)	47 (41.6)	45 (40.2)	0.87
Crowding^a^	2.2 (1.3)	2.0 (1.0)	0.35
Possessions^b^	8.9 (2.4)	8.9 (2.6)	0.98
**Child behavior in preschool**			
**Structured observations of child behavior**			
Aggressive/destructive behavior, *median (range)*^c^	12 (0–50)	13 (0–45)	0.53
Disruptive behavior, *median (range)*^d^	32 (3–89)	32 (6–98)	0.99
**Rating scales of child behavior**^**e**^			
Conduct problems	2.70 (0.85)	2.81 (0.85)	0.33
Activity level	3.32 (0.73)	3.19 (0.67)	0.08
On-task behavior	4.95 (0.87)	4.85 (0.84)	0.25
Follows rules and expectations	4.75 (0.72)	4.63 (0.67)	0.17
**Teacher-reported child behavior**			
Conduct problems (SESBI intensity scales)	154.29 (44.38)	152.45 (31.96)	0.86
Clinical range for conduct problems at school: *n*(%)^f^	60 (53.1)	63 (56.3)	0.64
Prosocial skills (SDQ)	5.30 (2.31)	5.49 (2.32)	0.50
**Parent-reported child behavior**			
Conduct problems (ECBI intensity scales)	120.05 (22.66)	119.83 (24.26)	0.90
Clinical range for conduct problems at home: *n*(%)^g^	42 (37.2)	35 (31.3)	0.35
Prosocial skills (SDQ)	7.33 (2.23)	7.82 (1.87)	0.07
**Children evaluated in grade one**	**Intervention** ***n=181***	**Control*****n=183***	***P***-value^*^
**Child and family characteristics**			
Child age (in years)	6.91 (0.39)	6.90 (0.38)	0.73
Child sex: *n* (%) boys	110 (60.1)	102 (56.4)	0.47
Family on PATH (cash-transfer) programme *n* (%)	19 (10.5)	22 (12.0)	0.53
Caregiver age	34.04 (9.29)	33.98 (9.85)	0.95
Caregiver finished secondary school *n* (%)	83 (46.1)	88 (49.2)	0.60
Father lives with child *n* (%)	69 (38.3)	56 (33.0)	0.16
Crowding *median (range*^a^	1.6 (0.4–6.0)	1.7 (0.4–8.0)	0.71
Possessions^b^	8.68 (2.32)	8.35 (2.49)	0.19
**Teacher/classroom characteristics**			
Teacher sex: *n* (%) female	180 (99.4)	183 (100)	0.32
Number of years teaching	16.48 (11.38)	17.57 (11.76)	0.37
Number of years teaching at current school	11.13 (9.23)	11.51 (9.54)	0.70
Teacher qualified *n* (%)	175 (96.7)	175 (95.6)	0.26
Teacher has early childhood teaching qualification: *n* (%)	66 (36.5)	76 (41.5)	0.37
Number of children in class	31.00 (7.52)	31.45 (6.38)	0.55

* *t-tests were used for normally distributed continuous variables, Mann-Whitney tests were used for continuous variables that were not normally distributed, Chi-square analyses were used for categorical variables. SESBI, Sutter-Eyberg Student Behavior Inventory; SSBS, School Social Behavior Scales; ECBI, Eyberg Child Behavior Inventory; SDQ, Strengths and Difficulties Questionnaire*.

a *Number of people per room*.

b *Number of possessions from a list of 15 items; stove, fridge, washing machine, sofa or soft chair, mobile phone, landline, radio, CD player, TV, cable TV, DVD player, computer, bicycle, motorbike, motor car*.

c *Counts over 12 5-min observation intervals conducted over 2 school days*.

d *Instantaneous sampling at 15 s intervals over a total of 1 h of observation over 2 school days (max = 240)*.

e *Mean of 12 ratings conducted after each 5-min observation periods on a scale of 0–7, where 0, low; 7, high*.

f *Above cut-off (>150) on SESBI intensity scale*.

g *Above cut-off (>130) on ECBI intensity scale*.

### Effect of Intervention

Raw scores for all outcomes are shown in Table 2 with the significance of unadjusted intervention effects. Table 3 shows the intervention effects using multi-level linear regression analyses. Benefits of the preschool teacher-training intervention were found for children's academic achievement (ES = 0.23), oral language (ES = 0.28), self-regulation (ES = 0.25), and school attendance (ES = 0.30) in grade one of primary school. No significant benefits were found for observed conduct problems (ES = −0.13), or parent-reported conduct problems (ES = 0.10) and prosocial skills (ES = 0.06). However, teacher-reported conduct problems (ES = −0.16) and social skills (ES = 0.19) were significant at *p* < 0.1.

**Table 2:** Raw data for child outcomes in grade one of primary school by study group. Values are Mean (SD) unless otherwise stated.

	**Intervention**	**Control**	*P*-value^*^
	***n = 181***	***n = 183***	
**Structured observations of child behavior**			
Aggressive/destructive behavior, *median (range)*^a^	6 (0–40)	6 (0–31)	0.19
Disruptive behavior, *median (range)*^b^	23 (0–93)	25 (2–99)	0.07
**Rating scales of child behavior, mean (SD)**^**c**^			
Conduct problems	2.01 (0.68)	2.07 (0.65)	0.38
Activity level	2.83 (0.032)	2.87 (0.36)	0.24
On-task behavior	5.04 (0.93)	4.91 (0.87)	0.19
Follows rules and expectations	5.40 (0.67)	5.31 (0.69)	0.21
**Teacher-reported child behavior**			
Conduct problems (SESBI frequency scales)	117.96 (42.38	126.07 (47.80)	0.09
Social skills (SSBS)	108.65 (22.76)	103.45 (23.08)	0.03
**Parent-reported child behavior**^**d**^			
Conduct problems (ECBI frequency scales)	116.28 (25.78)	115.03 (23.74)	0.63
Prosocial skills (SDQ)	9 (1–10)	9 (3–10)	0.81
**Academic achievement, language, and self-regulation**			
Letter-word identification	24.37 (8.97)	22.18 (7.96)	0.01
Reading comprehension	10.59 (5.04)	9.52 (4.50)	0.03
Spelling	19.15 (4.82)	17.79 (4.47	0.006
Maths calculation, *median (range)*	5 (0–12)	5 (0–11)	0.002
Maths reasoning, *median (range)*	25 (2–31)	25 (0–31)	0.005
Receptive language (following directions)	21.01 (8.24)	18.78 (8.55)	0.01
Expressive language (story recall)	23.53 (14.75)	20.14 (13.21)	0.02
Self-regulation, *median (range)*^e^	26 (5–30)	26 (8–30)	0.02
**School attendance**			
School attendance, *median (range)*^f^	94.44 (35.85–100)	89.47 (25.33–100)	<0.0001

* *t-tests were used for normally distributed continuous variables, Mann-Whitney tests were for continuous variables that were not normally distributed. SESBI, Sutter-Eyberg Student Behavior Inventory; SSBS, School Social Behavior Scales; ECBI, Eyberg Child Behavior Inventory; SDQ, Strengths and Difficulties Questionnaire*.

a *Counts over 1 h*.

b *Instantaneous sampling at 15 s intervals over a total of 1 h of observation (max=240)*.

c *Mean of 12 ratings conducted after 5-min observation periods on a scale of 0–7, where 0, low; 7, high*.

d *For parent-reported outcomes: n = 180 intervention, 179 control*.

e *Sum of 10 ratings of child behavior during the test on a scale of 0–4 (min=0, max=40)*.

f *Expressed as a percentage*.

**Table 3:** Multilevel regression analyses of the effect of the preschool teacher-training intervention on child outcomes in grade one of primary school.

	**Standardized Scores**^**a**^		**Effect size B (95% CI)^**b**^**	**ICC^**c**^**	***P*-value**
	**Intervention (*n* = 181)**	**Control (*n* = 183)**			
**Child behavior**					
Observed conduct problems^1^	−0.08 (0.99)	0.08 (1.01)	−0.13 (−0.32, 0.05)	0.12	0.16
Teacher-reported conduct problems^2^	−0.08 (0.93)	0.09 (1.05)	−0.16 (−0.35, 0.02)	0.03	0.09
Teacher-reported social skills^2^	0.12 (0.99)	−0.11 (1.00)	0.19 (−0.01, 0.38)	0.00	0.06
Parent-reported conduct problems^2^	0.03 (1.04)	−0.03 (0.96)	0.10 (−0.08, 0.30)	0.01	0.31
Parent-reported prosocial skills^2, 3^	−0.01 (1.07)	0.01 (0.93)	−0.07 (−0.27, 0.14)	0.02	0.52
**Child academic achievement, language, and self-regulation**					
Academic achievement^4^	0.16 (1.02)	−0.15 (0.95)	0.23 (0.04, 0.42)	0.28	0.02
Oral language^5^	0.16 (1.00)	−0.15 (0.95)	0.28 (0.08, 0.48)	0.13	0.006
Self–regulation^2, 3^	0.14 (0.94)	−0.13 (1.04)	0.25 (0.07, 0.43)	0.03	0.007
**Child attendance**					
Child attendance^2, 3^	0.17 (1.02)	−0.16 (0.97)	0.30 (0.10, 0.49)	0.09	0.003

a *Mean (SD)*.

b *Regression coefficient (95% confidence interval), expressed as standardized scores*.

c *Intracluster correlation coefficient*.

1 *Factor score of structured observations of aggressive/destructive behavior and disruptive behavior, and ratings of child conduct problems, activity level, on-task behavior, and follows rules/expectations over 12 5-min observation intervals over 2 days of observation*.

2 *Standardized by subtracting the mean and dividing by the SD*.

3 *Normalized by squaring prior to standardizing*.

4 *Factor score of Letter-Word Identification, Passage Comprehension, Spelling, Maths Calculation, and Maths Reasoning*.

5 *Factor score of Following Directions (receptive language) and Story Recall (expressive language)*.

*All regressions controlled for child age and sex, dummy variables for data collectors, dummy variables for year of data collection, and a dummy variable for in original study sample or not as fixed effects, and school and classroom as random effects*.

## Discussion

To our knowledge, this is the first trial of a preschool teacher-training, violence-prevention program from LMIC with follow-up measures of child outcomes when children have transitioned to primary school. The intervention involved training teachers in classroom behavior management and in how to promote child social-emotional competence through everyday teaching and learning activities. In this study, although we implemented a universal preventative intervention, we recruited children with the highest levels of conduct problems in preschool in the evaluation sample. We found significant benefits to: (1) direct tests of child academic achievement and oral language, (2) tester ratings of self-regulation (including child attention and impulse control), and (3) child school attendance from school records. No significant benefits were found for child conduct problems and social skills at home and at school.

We report effect sizes between 0.13 and 0.25 on child behavior at school, between 0.23 and 0.28 on child outcomes on an academic achievement test, and 0.30 on school attendance. These effect sizes are of a similar level of magnitude to those reported by meta-analyses of longer-term effects from universal, school-based, social, emotional, and behavioral programs that have largely been implemented in primary schools in high-income countries. These meta-analyses report effect sizes between 0.07 and 0.33 for children's social-emotional skills, conduct problems, prosocial behaviors, and academic achievement, with strongest effects for academic achievement (Sklad et al., 2012; Taylor et al., 2017). We are aware of only two previous studies, both conducted in the US, that investigated the longer-term effects of such programs implemented in preschool settings. These studies reported sustained benefits to child behavior, academic achievement and/or executive function (Zhai et al., 2012; Bierman et al., 2014; Sasser et al., 2017; Welsh et al., 2020), especially for children with poorer baseline functioning (Sasser et al., 2017) and for children who subsequently attended higher-quality schools (Zhai et al., 2012). However, these US programs were resource intensive (e.g., incorporating teacher mental health, extensive in-class support, services for high-risk children, use of a structured social-emotional curriculum, and/or an additional intervention component targeting child preacademic skills) (Bierman et al., 2008a; Raver et al., 2008), and hence would be unlikely to be affordable in LMIC contexts. Our intervention was teacher-focussed (Baker-Henningham et al., 2012; Baker-Henningham and Walker, 2018), and benefits to parent-reported child behavior were small at post-intervention (ES = 0.22), and there were no longer-term benefits to child behavior at home. For sustained gains to child behavior at home, a parent-training component is likely to be necessary (Bierman et al., 2015).

There are two main potential pathways for the effect of the intervention on child achievement, language, self-regulation, and attendance. Firstly, benefits post-intervention may have led to sustained benefits in primary school. For example, benefits to child attendance were found in preschool and these benefits were sustained in primary school, possibly due to increased parent interest and involvement in their child's schooling and/or children's increased bonding to school (Hawkins et al., 1992; O'Donnell et al., 1995). Although we did not measure child pre-academic, language, and self-regulation skills in preschool, it is possible that these skills were also improved at post-intervention (Raver et al., 2011), with benefits sustained in grade one. Secondly, gains to specific skills and behaviors at post-intervention may mediate the effect of intervention on other aspects of child functioning in primary school. That is, children's later attainments may build on earlier skill development (Heckman, 2006). For example, the benefits to child academic achievement and language skills may have been mediated by the gains to child behavior post-intervention (Nix et al., 2013). In addition, through the teacher-training program, preschool teachers were trained to provide a positive, structured classroom environment, with clear rules and expectations, and with behavioral supports to help children meet these expectations. These teacher behaviors promote the development of children's self-regulation skills, and in two recent Jamaican studies, we have found benefits to child self-regulatory competencies from training teachers in classroom behavior management (Baker-Henningham et al., 2019, 2021). Self-regulation has been shown to predict longer-term gains to child outcomes in other studies (Bierman et al., 2008b; Raver et al., 2011). Benefits to school attendance may have also mediated the effect of the intervention on child academic skills (Gottfried, 2010).

Grade one classrooms in inner-city primary schools in Kingston are characterized by low levels of emotional support, frequent use of harsh punishment by teachers, low levels of class-wide child prosocial behavior, and relatively high levels of class-wide child aggression (Baker-Henningham et al., 2019). The lack of a nurturing classroom environment and exposure to peers displaying aggressive behaviors and poor social skills may make it difficult for children to sustain gains to their behavior in this new context (Zhai et al., 2012; Wolf, 2019). It is perhaps surprising, that despite these non-sustaining environments, benefits to child functioning across multiple domains were found. It is possible that larger benefits to child outcomes, including child conduct problems and social skills would be found if training was also provided for teachers in the early primary grades to ensure a consistent approach as children transition from preschool to formal schooling.

The strengths of the study include: (1) use of multiple informants to measure child behavior, including independent observations, teacher, and parent report (Scott, 2001), (2) use of direct tests of child school achievement and language skills, and tester ratings of child self-regulation, (3) all measurements administered by masked assessors, (3) good psychometric properties of the outcome measures, and (4) low attrition of the original study sample with over 95% of children followed up in primary school. The study also has limitations. We had limited power to detect small effects and it is possible that a larger sample size may have shown significant effects on child behavior at school, especially for teacher-reported outcomes which were significant at *p* < 0.1. Some factors may limit the generalisability of the results. Children with high levels of conduct problems who had poor preschool attendance were ineligible to participate in the evaluation. However, only 25 children were excluded for poor attendance, <7% of the sample. Preschools in the original trial were selected based on the number of classrooms and number of children per class due to logistical reasons relating to training, measurement, and identification of high-risk children. However, as the intervention involves training all teaching staff, we anticipate benefits for children attending schools with different numbers of classrooms and/or smaller class sizes. We were unable to conduct longitudinal analyses to examine mediators of intervention effectiveness as over 40% of the children included in this study were not evaluated in preschool. As we only recruited children with heightened levels of conduct problems at baseline, we do not know whether the intervention benefited all children, or whether benefits were concentrated in children at high-risk.

In conclusion, we found that a low-cost, scalable teacher-training, violence-prevention program led to benefits across multiple outcomes in grade one primary school for children with high initial levels of conduct problems. Future research is required to examine whether the intervention benefits children with low-to-moderate levels of conduct problems, in addition to those at heightened risk and whether benefits are sustained over the longer-term. It is also important to investigate the potential for cumulative effects to child functioning from training preschool and primary school teachers and from combining the teacher-training with a complementary parent-training program to promote an integrated approach across contexts.

## Data Availability Statement

The raw data supporting the conclusions of this article will be made available by the authors, without undue reservation.

## Ethics Statement

The studies involving human participants were reviewed and approved by University of the West Indies Ethics Committee, Mona, Kingston, Jamaica. Written informed consent to participate in this study was provided by the participants' legal guardian/next of kin.

## Author Contributions

HB-H and SW contributed to the conceptualization of the study and funding acquisition. HB-H and YS contributed to project administration. YS, TF, and HB-H contributed to investigation. TF and YS were responsible for data curation. HB-H and TF were responsible for data analysis. HB-H was responsible for writing the original draft. All authors reviewed and edited the manuscript.

## Conflict of Interest

The authors declare that the research was conducted in the absence of any commercial or financial relationships that could be construed as a potential conflict of interest.
