# Supplementary material for: Effects of a Teacher-Training Violence Prevention Program in Jamaican Preschools on Child Behavior, Academic Achievement, and School Attendance in Grade One of Primary School: Follow up of a Cluster Randomized Trial
Source: Front Psychol. 2021 Jun 3;12:652050. doi: 10.3389/fpsyg.2021.652050 (PMC8210533; doi:10.3389/fpsyg.2021.652050)
Supplement: Supplementary file 1 [file Data_Sheet_1.pdf]

Supplementary Material

Supplementary Table 1. Description of Child Outcomes

Outcomes	Measures Used
**Conduct Problems**	
Observed conduct problems	Observations over a total of 12 5-minute observation periods (totaling 1 hour of observation), including event sampling of aggressive/destructive (e.g. throwing objects, destroying property) behavior, scan sampling of disruptive behavior (e.g. yelling, out of seat) and four 7-point rating scales: conduct problems, activity level, on-task behavior, and follows rules and expectations.
Teacher-reported conduct problems	Measured using Sutter-Eyberg Child Behaviour Inventory (SESBI) frequency scale. The SESBI measures the frequency of child disruptive behaviors at school and includes 38 questions answered on a 7-point scale (1=never to 7=always). Example questions include: Loses his/her temper; Refuses to obey until threatened with punishment; Has difficulty accepting criticism or correction.
Parent-reported conduct problems	Measured using the Eyberg Child Behaviour Inventory (ECBI) frequency scale. The ECBI measures the frequency of child disruptive behaviors at home and includes 36 questions answered on a 7-point scale (1=never to 7=always). Example questions include: Loses his/her temper; Refuses to go to bed when you tell him/her; Complains or nags.
**Social Skills**	
Teacher-reported social skills	Measured using the School Social Behaviour Scales 2 (SBSS-2): Social Competence Scale. The SSBS-2 includes 32 questions measures on a 5-point scale (1=never to 5=frequently). Example questions include: Offers to help other students when needed; Listens to and carries out directions from teachers; Is invited by peers to join in activities.
Parent-reported social skills	Measured using the Strengths and Difficulties Questionnaire: Prosocial Scale. The prosocial scale consists of five questions answered on a 3-point scale (0=not true, 1=somewhat true, 2=certainly true). Example questions include: Shares readily with other children (treats, toys, pencils); Considerate of other people's feelings; Helpful if someone is hurt, upset or feeling ill.
**Academic Achievement**	
Reading	Measured using the Letter Word Identification and Passage Comprehension subscales from the Woodcock-Johnson III Tests of Achievement.<br>The letter-word identification test measures children's ability to identify letters and read single words of increasing difficulty. The Passage Comprehension Test measures reading comprehension and children are asked to supply the missing word in a sentence or paragraph.
Maths	Measured using the Calculation and Reasoning and Concepts subscales of the Woodcock-McGrew-Werder Mini-Battery of Achievement. The Reasoning and Concepts subscale measures children knowledge of mathematical concepts and vocabulary and analyzing and solving practical mathematical problems. Children answer the questions orally. The Calculation subscale is a written test that measures children's skills in performing basic mathematical operations such as addition, subtraction, multiplication and division.
Spelling	Measured using the Spelling subscale from the Woodcock-Johnson III Tests of Achievement. Children are asked to write letters followed by single words of increasing difficulty.
**Oral Language**	Measured using the Understanding Directions and Story Recall subscales from the Woodcock-Johnson III Tests of Achievement. Understanding Directions measures child receptive language. Children are asked to listen to and then point to objects in a series of pictures with the directions becoming more complex as the test progresses. Story Recall measures expressive language and children are asked to listen to and then retell increasingly complex stories.
**Self-Regulation**	Rated during the testing session for academic achievement and oral language using 10 4-point scales from the Preschool Self-Regulation Assessment. Five items measured attention (pays attention, careful, concentrates, daydreams, distracted) and five items measured impulse control (thinks and plans, refrains from touching test materials, doesn't interrupt tester, difficulty waiting, remains in seat).
**School Attendance**	Collected from school records. We used the average attendance over term 1 and term 2 in grade one of primary school.

Supplementary Table 2: Internal reliability and test-retest of child outcome measures

	Internal Reliability	Test-Retest (ICC)
Teacher reported child behaviour		
Sutter-Eyberg Student Behaviour Inventory	0.97	0.95
School Social Behaviour Scales 2 (Social Skills Scale)	0.95	0.90
Parent reported child behaviour		
Eyberg Child Behaviour Inventory	0.83	0.93
Prosocial skills (Strengths and Difficulties Questionnaire)	0.71	0.75
Child academic achievement and language tests and tester ratings of self-regulation		
Letter-word identification	-	0.98
Reading comprehension	-	0.98
Mathematical reasoning	-	0.95
Maths calculation	-	0.99
Spelling	-	0.94
Story recall	-	0.75
Following directions	-	0.88
Self-regulation	0.88	0.85

Test-retest was conducted over 2 weeks for all measures: n=20 for teacher report measures & child tests, n=18 for parent report measures. ICC: intraclass correlation coefficient

Supplementary Table 3: Interobserver reliabilities for observed conduct problems in primary school

	Training Reliabilities[1] ICC (95% CI)[3]	Ongoing Reliabilities[2] ICC (95% CI)[3]
Structured observations of child behaviour		
Aggressive/destructive behaviour	0.90 (0.80-0.96)	0.92 (0.87-0.96)
Disruptive behaviour	0.94 (0.88-0.98)	0.96 (0.93-0.98)
Rating scales of child behaviour		
Conduct problems	0.95 (0.81-0.97)	0.94 (0.89-0.97)
Activity	0.91 (0.82-0.96)	0.84 (0.71-0.92)
On-task behaviour	0.95 (0.91-0.98)	0.93 (0.85-0.99)
Follows rules	0.95 (0.89-0.98)	0.97 (0.94-0.99)

[1]n=20 observations between trainer and each observer and minimum of 15 between each observer and other observers. [2]minimum of 10% of all observations between senior research assistant and each observer. [3]ICC: intraclass correlation coefficient

Supplementary Table 4: Factor analyses of observed child behavior, academic achievement, and oral language in primary school

**Child Observed Behaviour**	**Factor loading**
Follows rules	-0.903
Disruptive behavior	0.805
Conduct problems	0.801
Activity	0.797
Aggression	0. 778
On-Task behaviour	-0.674
Variance explained, %	*63.31*
**Academic Achievement Tests**	**Factor loading**
Letter-word ID	0.936
Spelling	0.930
Reading comprehension	0.900
Calculation	0.794
Maths	0.783
Variance explained, %	*75.92*
**Oral language tests**	**Factor loading**
Following directions	0.831
Story recall	0.831
Variance explained, %	*69.09*

Supplementary Table 5 Child and family characteristics and baseline values of child behavior by loss at follow-up for children evaluated in preschool. Values are Mean (SD) unless otherwise stated

	**Lost** *n=11*	**Found** *n=214*	**P-Value**
Child characteristics			
Child age (in years)	3.73 (0.60)	4.23 (0.87)	0.02
Child sex: n (%) boys	4 (36.4)	134 (62.6)	0.08
Family characteristics			
Caregiver age	30.91 (19.96)	29.64 (11.29)	0.73
Caregiver finished high school n (%)	7 (63.6)	125 (58.4)	0.73
Father lives with child n (%)	6 (54.5)	126 (59.2)	0.76
Crowding	2.28 (0.91)	2.07 (1.17)	0.55
Possessions	8.00 (2.10)	8.94 (2.54)	0.23
Structured observations of child behaviour			
Aggressive/destructive behaviour, *median (range)*	16 (0-50)	12 (0-48)	0.76
Disruptive behaviour, *median (range)*	31 (6-89)	33 (3-98)	0.78
Rating scales of child behaviour			
Conduct problems	2.64 (1.02)	2.76 (0.83)	0.64
Activity level	3.27 (0.71)	3.26 (0.66)	0.98
On-task behaviour	4.98 (0.95)	4.92 (0.85)	0.81
Follows rules and expectations	4.78 (0.88)	4.68 (0.68)	0.62
Teacher-reported behaviour			
Conduct problems (SESBI frequency scales)	149.27 (27.36)	154.09 (39.30	0.70
Prosocial Skills (SDQ)	5.36 (2.06)	5.43 (2.32)	0.93
Parent-reported behaviour			
Conduct problems (ECBI frequency scales)	121.09 (20.60)	119.17 (23.86)	0.80
Prosocial skills (SDQ)	6.73 (2.83)	4.23 (0.87)	0.33
